# Altered Microcirculation in Alzheimer’s Disease Assessed by Machine Learning Applied to Functional Thermal Imaging Data

**DOI:** 10.3390/bioengineering9100492

**Published:** 2022-09-21

**Authors:** David Perpetuini, Chiara Filippini, Michele Zito, Daniela Cardone, Arcangelo Merla

**Affiliations:** 1 Department of Neuroscience and Imaging, University “G. D’Annunzio” of Chieti-Pescara, 66100 Chieti, Italy; 2 Department of Medicine and Science of Ageing, University “G. D’Annunzio” of Chieti-Pescara, 66100 Chieti, Italy; 3 Department of Engineering and Geology, University “G. D’Annunzio” of Chieti-Pescara, 66100 Chieti, Italy; 4 Next2U s.r.l., 65127 Pescara, Italy

**Keywords:** Alzheimer’s disease, infrared thermography (IRT), microcirculation, autonomic impairments, frequency bands analysis, machine learning (ML)

## Abstract

Alzheimer’s disease (AD) is characterized by progressive memory failures accompanied by microcirculation alterations. Particularly, impaired endothelial microvascular responsiveness and altered flow motion patterns have been observed in AD patients. Of note, the endothelium influences the vascular tone and also the small superficial blood vessels, which can be evaluated through infrared thermography (IRT). The advantage of IRT with respect to other techniques relies on its contactless features and its capability to preserve spatial information of the peripheral microcirculation. The aim of the study is to investigate peripheral microcirculation impairments in AD patients with respect to age-matched healthy controls (HCs) at resting state, through IRT and machine learning (ML) approaches. Particularly, several classifiers were tested, employing as regressors the power of the nose tip temperature time course in different physiological frequency bands. Among the ML classifiers tested, the Decision Tree Classifier (DTC) delivered the best cross-validated accuracy (accuracy = 82%) when discriminating between AD and HCs. The results further demonstrate the alteration of microvascular patterns in AD in the early stages of the pathology, and the capability of IRT to assess vascular impairments. These findings could be exploited in clinical practice, fostering the employment of IRT as a support for the early diagnosis of AD.

## 1. Introduction

Alzheimer’s disease (AD) is a kind of dementia that mainly affects human memory abilities [1,2], and it is characterized by senile plaques, neurofibrillary tangles, and amyloid angiopathy [3]. The senile plaques are mainly constituted of amyloid beta peptide, which is generally associated with cerebrovascular alterations, indicating that vascular damage could be involved in the development of AD and not only in the pathogenesis of vascular dementia [4].

In addition, although AD is mainly considered a neuronal disease, peripheral alterations have been found, particularly in the hemostatic cells such as platelets. Several authors, in fact, reported platelet abnormalities in protein kinase C (PKC)- and phospholipase C (PLC)-dependent signal transduction [5], serotoninergic and glutamatergic pattern receptors [6], and membrane fluidity [7]. Of note, it has been demonstrated that AD is specifically associated with abnormalities in the patterns of platelet amyloid precursor protein forms [8,9]. Furthermore, altered peripheral endothelial cell functions have been assessed [10]. For instance, Khalil et al. demonstrated altered peripheral endothelial vascular responses in AD [11] by employing laser doppler flowmetry, a technique that allows to continuously and non-invasively monitor changes in microvascular perfusion, with regard to the relative changes of blood volume and velocity [12].

Moreover, hemodynamic alterations in AD have been investigated through photoplethysmography (PPG), an optical technique able to measure the blood volumetric oscillations in vessels related to heart rate [13]. Particularly, Iwamoto et al. assessed a relationship between the decreased distensibility in the aortic wall and the white matter lesions in AD [14], and Gwak et al. developed a method to support a mild cognitive impairment (MCI) diagnosis based on PPG [15].

Together with peripheral dysfunctions and central nervous functioning decline, subtle autonomic abnormalities in AD have been investigated in several studies [16,17,18] demonstrating pathological changes in the autonomous nervous system (ANS) in AD. For instance, an increase in cold intolerance was found to be associated with the course of the disease, highlighting both pathophysiological changes and adaptive behavior [19]. This demonstrates that this symptom is not only related to the decline of neural mechanisms involved in autonomic thermoregulatory strategies but also to behavioral thermoregulation, whose neural and physiological substrates need to be further investigated [20]. Concerning ANS impairments, other studies have focused on core body-temperature modifications in AD [21,22], or alterations of the circadian rhythm [23]. Moreover, differences between AD patients and healthy controls (HCs) have been investigated through infrared thermography (IRT) [24,25] during the execution of cognitive tasks commonly used for an AD diagnosis. These studies reported an altered autonomic response in AD patients with respect to HCs, which could be related to impairments of the ANS or abnormal emotional responses [26].

IRT is a technology able to measure the cutaneous temperature of an individual in a contactless manner [27]. The temporal modulation of the cutaneous temperature of responsive regions (e.g., nose tip, chin, and perioral regions) have been demonstrated as sensitive to the autonomic state of an individual [28]. Hence, given its sensitivity in detecting stress, anxiety, fatigue, and emotions [29,30,31,32,33], IRT has been widely employed for monitoring the psychophysiological status of a subject and for affective computing applications.

The data analysis for IRT signals is generally based on time-domain data analysis (e.g., differential or slope analysis) or frequency-based analysis [34]. Moreover, approaches of machine learning (ML) and deep learning (DL) have been proposed for the IRT signals’ data analysis in order to increase the capability of this technique to assess pathologies and autonomic activations [35,36]. ML is a field of applied statistics that uses multivariate approaches for prediction purposes [37], often used in biomedical applications to classify patients from HCs by employing features computed on physiological signals. The ML approach is accompanied by a cross-validation procedure that reduces the overfitting effect of the results, hence improving the generalization performance of the model [38].

Several studies have employed ML algorithms to discriminate HCs from AD, mainly relying on the analysis of central nervous system activity. Specifically, the classification of AD patients from HCs and MCI has been based on electroencephalography (EEG) [39], functional magnetic resonance imaging (fMRI) [40,41,42], and functional near-infrared spectroscopy (fNIRS) [43]. Of note, a recent review by Tanveer et al. [44] describes how multiple ML approaches (e.g., SVM, artificial neural network (ANN), DL, and ensemble methods) have been proposed so far to discriminate AD from HCs and MCI; however, studies regarding the employment of ML techniques for IRT data analysis to discriminate AD from HCs are missing.

In this study, IRT was employed to detect microvascular dysregulations in early AD patients. Specifically, the frequency-domain analysis of IRT signals acquired during rest was performed to feed a cross-validated ML framework. In detail, the power of the thermal signal collected over the nose tip in specific frequency bands [45,46] was computed: neurogenic band (0.02–0.04 Hz), associated with neuronal activity; myogenic band (0.04–0.15 Hz) indicative of the activity of the smooth muscles of arterioles; respiratory band (0.15–0.5 Hz), suggestive of the breathing status; cardiac band (0.5–1 Hz), indicative of the heart functioning. The average power of the most indicative frequency bands was used as input for the ML algorithms. A leave-one-out cross-validation was implemented to preserve the generalization performance of the classification.

The novelty of this study consists in the assessment of microvascular dysregulation in early AD patients during a resting state through IRT. In fact, previous studies have assessed abnormal thermal modulations in early AD patients during the execution of cognitive tasks, but, to the best of the Authors’ knowledge, this is the first paper evaluating altered spontaneous skin thermal modulation in early AD patients. The advantages of employing IRT, with respect to other techniques able to measure microvascular alterations, rely on its contactless and non-invasive features, hence being suitable for clinical screening finalized for an early diagnosis of this kind of dementia.

## 2. Materials and Methods

### 2.1. Participants

Twenty-six participants were enrolled in the study. The study sample was composed of 11 AD patients (mean age ± standard deviation (SD): 71.4 ± 4.7 years; M/F: 6/5) and 15 HCs (mean age ± SD: 69.3 ± 5.8 years; M/F: 10/5). The AD patients had a diagnosis of mild, probable Alzheimer’s disease, as defined by the Diagnostic and Statistical Manual of Mental Disorders, 5th edition (DSM-5). The exclusion criteria of the study were: vascular dementia, behavioral or psychiatric disorders, brain lesions or a history of stroke or traumatic brain injury, and moderate cognitive impairment (Mini-Mental State Examination, MMSE < 20/30) [47]. Moreover, participants with circulatory diseases that could affect the cutaneous temperature were not enrolled in the study. Of note, MCI patients also were not included in the study. During the experimental session, participants sat comfortably on a chair and were asked to stay as still as possible with their eyes closed and to not think about anything specific during the recordings; hence, 5 min of resting-state recordings were performed. Standard guidelines for thermal measurements were followed during the experimental session [48]. In particular, the measurements were performed in a thermoneutral environment to avoid thermoregulatory-induced alterations; moreover, patients had a period of 15 min of acclimation before the session, to reach thermal equilibrium with the environment [49]. Furthermore, in order to prevent potential effects related to circadian-rhythm variations, all sessions were scheduled at the same time of day [50].

### 2.2. IRT Instrumentation and Thermal Signals Data Analysis

The facial temperature was recorded through a digital thermal infrared camera, the FLIR SC660 (FLIR, Wilsonville, OR, USA) (640 × 480 bolometer FPA, sensitivity/noise equivalent temperature difference: <30 mK @ 30 °C, field of view: 24° × 18°). The camera was placed at a distance of 60 cm from the participant and pointed toward his/her face. The sampling frequency was 10 Hz. In order to minimize the potential drift/shift of the sensor’s response and optical artifacts, the camera was blackbody calibrated.

**Figure 1:**
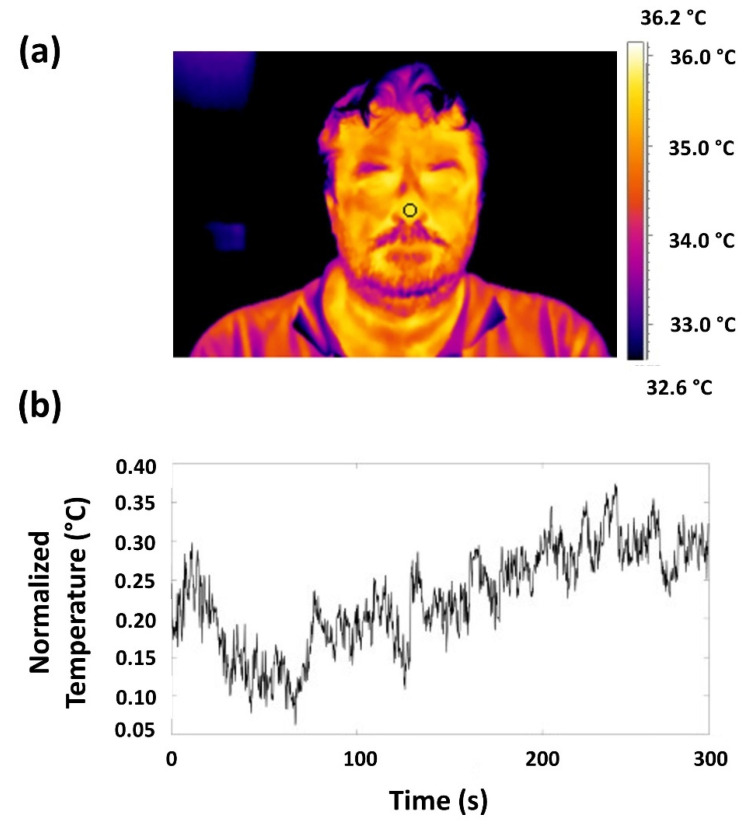
(**a**) Thermogram of a representative participant and ROI placement over the nose tip; (**b**) temperature oscillations over the selected ROI.

The quality of recorded thermal signals was first checked by visual inspection, no video was rejected. One region of interest (ROI) was selected on the nose tip of each subject (Figure 1a). This ROI moved in solidarity with the nose tip within each frame of the video through the employment of a tracking algorithm [51]. When the tracking algorithm failed (e.g., due to too wide a head rotation), the contaminated samples were substituted with the mean value of six samples before and after the motion period. The tracking algorithm allowed us to obtain the time course of the temperature of the ROI selected (Figure 1b).

The power spectral density (PSD) of the thermal time-course was computed. The area under the curve of the PSD was evaluated for the neurogenic band (0.02–0.04 Hz), the myogenic band (0.04–0.15 Hz), the respiratory band (0.15–0.5 Hz), and the cardiac band (0.5–1 Hz).

### 2.3. Multivariate Data-Driven Analysis and Statistical Inference

Supervised ML refers to the learning of a set of rules from instances aiming to define functions to link an input to an output. The model is usually defined on a training dataset, and tested and validated on data not used for the training process. This approach allows us to investigate the accuracy of the learned function and the generalization performance of the model [52]. In this study, four kinds of classifiers were compared: the Nearest Neighbor (kNN), Ensemble Classifier, Decision Tree Classifier (DTC), and Support Vector Machines (SVM).

The kNN classifiers are non-parametric classifiers that predict the label of the test samples by relying on the predominant class of its k most similar training samples in the feature space [53]. In this study, fine, medium, and coarse kNN models were investigated.

The Ensemble classifiers employ several learning algorithms to reach higher classification performance with respect to those obtained by the single constituent learning algorithms [54]. Bagged trees and the subspace discriminant were considered in this work.

The SVM classifiers estimate the optimal separating boundaries between groups for solving a constrained quadratic optimization problem. The model could include several degrees of nonlinearity, employing different kernel functions. In this study, linear, quadratic, cubic, and radial basis function (RBF) kernels were considered. Specifically, the RBF function is characterized by the following formula for two vectors, u and v:

RBF (u, v) = exp(−γ‖u−v‖^2^)

where γ is a hyperparameter used as a similarity measure between two data points. In this study, γ was set as 0.5.

DTC is a supervised ML algorithm that uses a set of rules to make decisions, employing dataset features to create yes/no questions and continually splitting the dataset until it is possible to isolate all data points belonging to each class. This process allows us to organize the data in a tree structure, in fact, each question adds a node and branches to the tree.

In order to avoid the overfitting effect that could derive from a high number of features with respect to the sample numerosity, only 2 metrics (i.e., power at the cardiac and respiratory frequency bands) were considered (i.e., 2 input features). The input metrics were normalized (z-score). The choice of these metrics was driven by an explorative inferential statistic finalized to assess the differences between the two groups. The normality of the distribution of the metrics was checked by Shapiro-Wilk’s normality test. Since all the data met the assumption of normality, the statistical differences between the two groups were assessed by a parametric test (i.e., *t*-test).

The models were trained on all subjects except one and tested on the remaining subject in an iterative manner (i.e., leave-one-out cross-validation) [38,55]. The out-of-training-sample output of the regression was investigated through a receiver operating characteristic (ROC) analysis, associating a labeling value to AD and HCs, to investigate the capabilities of the developed models to discriminate between the two groups.

**Figure 2:**
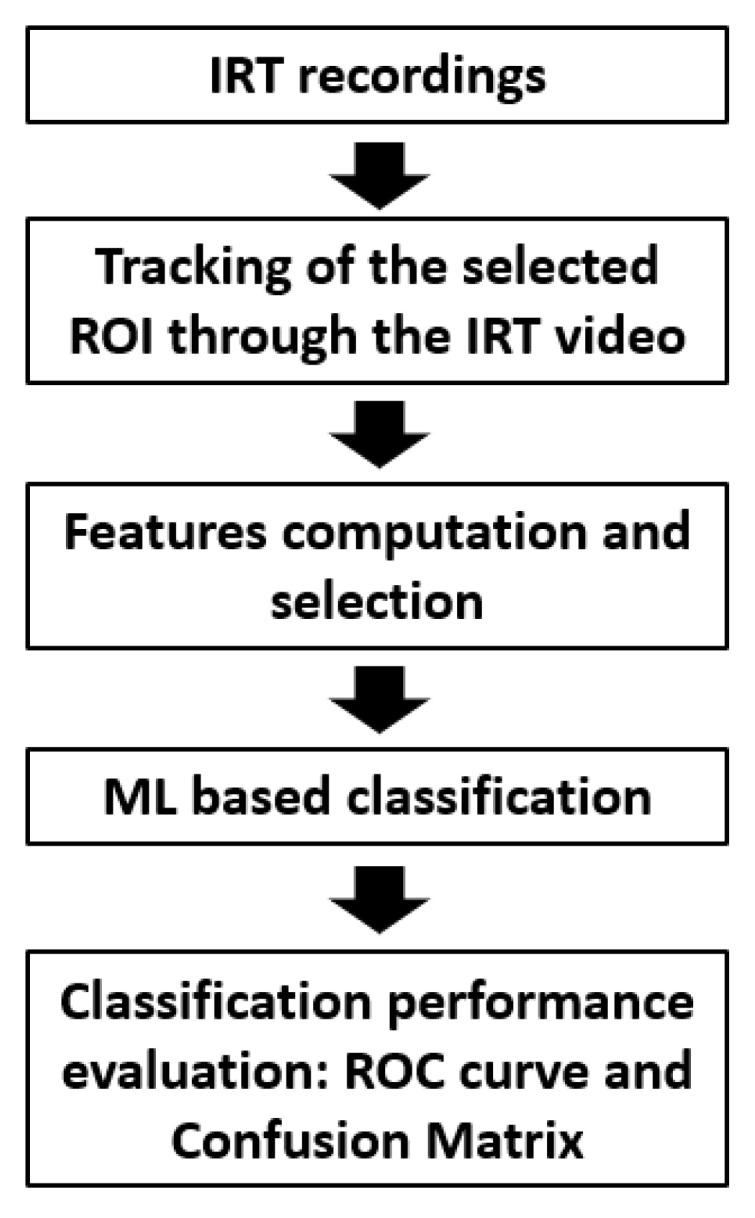
Flowchart describing the processing pipeline: data acquisition, processing, and ML-based classification.

A flowchart describing the processing pipeline is reported in Figure 2.

Concerning the computation efficiency of the proposed method, tracking the ROI through the IRT video required an average time of 0.057 s/frame. Considering the length of the IRT recordings of 3000 frames (duration: 5 min; sample frequency: 10 Hz), the average computation time was 171 s. The evaluation of the PSD of the temperature time course for the feature extraction required 0.04 s. Finally, the classifier that should be run for the single participant required a computation time of 0.275 s. Notably, the analysis was performed using a PC with Windows 10Pro (Intel(R) Core (TM) i7-7700; CPU @ 3.60 GHz; RAM: 16 GB).

## 3. Results

### 3.1. Inferential Statistics

**Figure 3:**
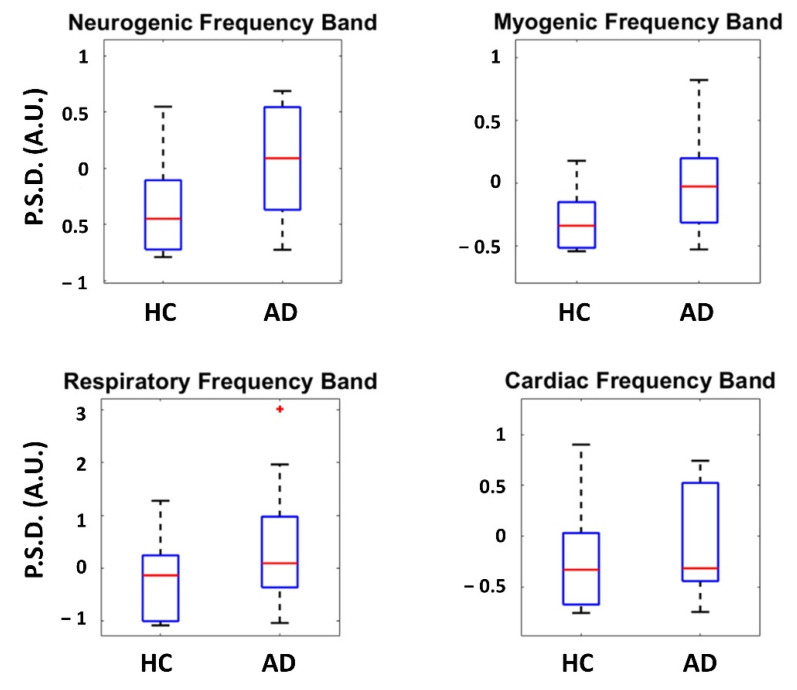
PSD evaluated in the considered frequency bands on the thermal signal collected from the nose tip. Each box shows the median and interquartile range, with the whiskers indicating the range of values. Data points beyond the whiskers are displayed as red ‘+’ in the figure.

Table 1 reports the results of the *t*-tests between AD and HCs for all the PSD evaluated in the considered frequency bands on the thermal signals collected from the nose tip. The inferential statistics did not show significant differences between AD and HCs. Figure 3 shows the boxplot associated with each metric evaluated for the two groups.

### 3.2. Machine Learning Analysis

The ML frameworks were implemented employing the PSD at the cardiac and respiratory frequency bands in the thermal signal acquired over the nose tip at rest since these two features were found to be more discriminative of the two groups by the inferential statistics analysis. The classification performances are reported in Table 2.

Considering the performances obtained in terms of accuracy, sensitivity, and specificity, the best models for discriminating AD and HCs are the DTC and the SVM with an RBF kernel.

**Figure 4:**
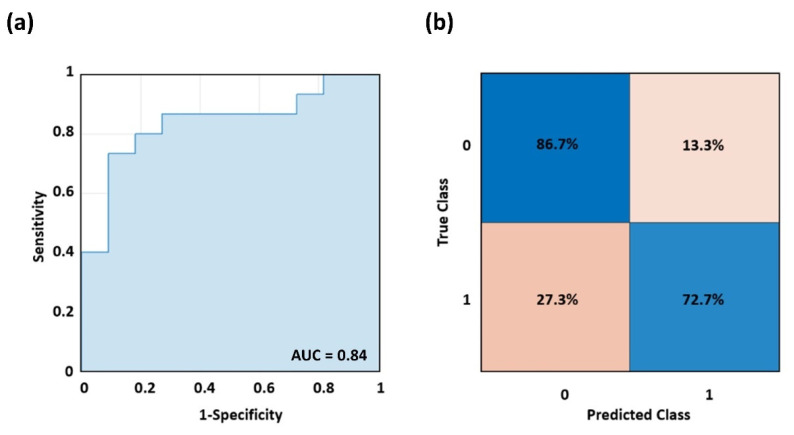
(**a**) ROC curve delivered by the cross-validated SVM classifier when discriminating AD and HCs, and (**b**) associated confusion matrix.

Specifically, the ROC analysis performed on the out-of-sample output of the SVM to classify the AD patients from HCs delivered an Area Under the Curve (AUC) of 0.84 (Figure 4a). The associated confusion matrix is reported in Figure 4b. The sensitivity of the model is 72.7% whereas the specificity is 86,7%, delivering an accuracy of 79.7%. The parameters of the SVM model were: γ = 0.5 and c = 1000, where γ is the inverse of the radius of influence of samples selected by the model as support vectors, and c trades off correct classification of training examples against the maximization of the decision function’s margin.

**Figure 5:**
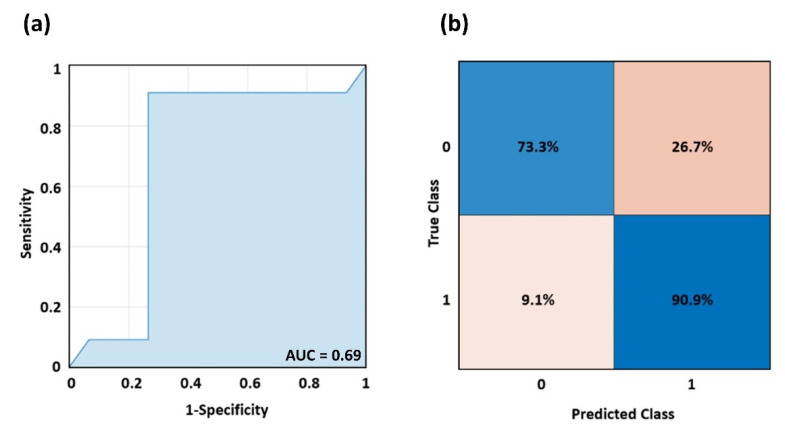
(**a**) ROC curve delivered by the cross-validated DTC when discriminating AD and HCs, and (**b**) associated confusion matrix.

Figure 5a reports the ROC curve delivered by the DTC model, with an AUC of 0.69. Figure 5b shows the relative confusion matrix, disclosing a sensitivity of 90.9% and a specificity of 73.3%, with an accuracy of 82.1%. The optimal DTC parameters were a max depth = 10 and a minimum samples leaf = 1, where the max depth indicates how deep the tree can be, and the minimum samples leaf is the minimum number of samples required to be at a leaf node.

Of note, the AUCs delivered by the two types of machinery do not exhibit statistical differences (z = −1.09; *p* = 0.275).

## 4. Discussion

AD is a kind of dementia that is denoted by a cognitive decline that can impair daily life [56]. Although the diffusion of this pathology, the physiological mechanisms underlying the etiology of the AD symptoms, are mainly unknown [57]. The AD diagnosis is currently performed by administering clinical cognitive tests able to assess the memory and visuo-spatial abilities of the patients. The diagnosis is usually confirmed by MRI imaging, which reveals deposits of the amyloid beta peptide. Moreover, it was demonstrated that advanced data analysis methods applied to signals collected by employing ecological neuroimaging tools (i.e., EEG and fNIRS) could support early AD diagnosis. In fact, these technologies could be easily introduced into clinical practice to assess the impaired brain functionality of AD patients, with respect to HCs during the administration of clinical tests [2]. Moreover, it was demonstrated that brain functionality is altered in AD patients in resting-state conditions, as seen by changes in the brain electrical activity modulations, cortical hemodynamics, and neurovascular coupling [58,59]. Of note, these techniques are able to assess abnormal brain activities and impaired resting-state oscillations. Furthermore, the synchronous measurements of EEG and fNIRS can deliver information on neurovascular coupling, the phenomena responsible for the blood-oxygenated-level-dependent (BOLD) effect. A disruption in neurovascular coupling could be related to alterations in the functionality of the brain vessels [60,61], or it could be plausible that the impaired brain microcirculation may trigger the pathology [62]. Moreover, an altered brain circulation could be indicative of a more generalized alteration of the circulatory system that also affects the peripheral vessels.

In this regard, several studies focused on the link between AD symptoms and the vascular state. Particularly, it was demonstrated that AD patients exhibit altered peripheral endothelial vascular activity [11], and the relationship between decreased distensibility of the aortic wall and white matter lesions in AD was assessed [14]. These studies suggested that as well as cognitive decline, AD is accompanied by disrupted brain circulation, and altered peripheral circulation could also be associated with dysfunctions of the ANS.

For instance, differences in ANS activation during the administration of clinical tests used for the diagnosis of pathologies have been assessed [25]. The findings demonstrated that during the execution of clinical tests, not only the cognitive functions involved but also the ANS plays a fundamental role, which could disclose differences between AD patients and HCs. Moreover, many authors investigated autonomic disorders in AD patients [18]. For instance, several studies focused on sleep-breathing-related disorders in AD patients identify obstructive sleep apnea as a potentially modifiable risk factor for AD [63]; furthermore, it has been demonstrated that continuous positive airway pressure treatment for sleep-breathing-related disorders may delay the progression of cognitive impairment in AD patients [64]. AD patients were found to exhibit an altered heart rate variability (HRV) with respect to HCs assessed through the low-frequency and high-frequency ratio (LF/HF). Particularly, the AD patients showed hyper-sympathetic activity during 5 min of rest in different positions (upright and supine posture) with respect to HCs [65]. Conversely, Mellingsæter and colleagues found a lower LF/HF ratio of the HRV in AD patients when compared to HCs during a head-up tilt test [66]. Furthermore, abnormal vasomotor sympathetic functions were revealed in AD patients as a reduced finger pulse amplitude during the Valsava maneuver, disclosing both parasympathetic and sympathetic vasomotor dysfunctions [17]. Notably, the latter finding could explain the variations in the circadian temperature [67] and thermoregulation [19], typical of AD. In fact, the vessel’s vasomotor regulation influences skin temperature, thus, this could be the physiological substrate of the altered cutaneous temperature oscillations assessed in this study. Moreover, it should be highlighted that impairments in the endothelium, typical of AD, can influence the vascular tone as well as in the small, superficial blood vessels [68].

The aim of this study was to assess microcirculatory dysfunctions in AD patients during a resting-state period of 5 min through facial IRT. In detail, the PSD of the thermal signal acquired on the nose tip was evaluated for the different frequency windows. The inferential statistic did not show significant differences between the two groups. Particularly, the cardiac and respiratory bands exhibited a tendency towards significance, whereas the other frequency bands were quite far from the level of significance accepted (*p* < 0.05). The results are in line with previous studies, demonstrating an altered HRV and breathing disorders in AD patients. However, with reference to the previous literature, a significant difference for the myogenic band was expected. The myogenic band is associated with the activity of the smooth muscles of arterioles, and hence could be indicative of impaired vasomotor functions. These findings could be related to the limited number of participants and/or to the anatomy of the nose tip. In fact, it is known that the main blood supply source of the nasal tip is the lateral nasal artery for most people [69], hence it could be plausible that the finding of this study is related to the ROI placement. However, it should be highlighted that in previous studies the nose tip has been proved to be highly indicative of ANS activity, highlighting differences between AD patients and HCs better than other facial regions [24,25]. Moreover, the nose tip could be sensitive to the convective movement of the air due to the breathing rate modulations, related to ANS activity. For this reason, the ROI was placed over the nose tip, and it was preferred to use only one ROI because of the limited sample size of the study. In fact, the employment of a reduced number of features to feed the multivariate ML approach could be useful to decrease the risk of overfitting when a limited number of participants are available.

Concerning the performances of the two best ML frameworks tested, it is possible to observe a non-statistically different AUC between the two methods, demonstrating a similar performance in accuracy. However, it should be highlighted that the SVM model delivers a higher specificity to AD with respect to DTC but the latter shows higher sensitivity. This finding suggests that the application of these models should be related to the aim of the applications; in fact, the DTC model should be preferred when high sensitivity is necessary, while the SVM should be employed when a large specificity to the pathology is needed.

Several studies have been performed so far to detect AD, relying on EEG, fMRI, computed tomography (CT) scans, positron emission tomography (PET), and fNIRS reaching performances of accuracy above 98%, as reported in the review by Alberdi et al. [70]. Of note, the introduction of deep learning approaches further improved the classification performances, reaching through the Convolutional Neural Network (CNN) applied to fMRI data accuracies above 99% [71,72]. These results demonstrate that CNN-based approaches outperformed conventional methods but require large training data sets to achieve optimal performance. Although the accuracy reached in the present study (~80%) is lower than those reported in the literature, it is worth highlighting that it could be ascribed to the limited sample size. Moreover, it should be stressed that the proposed method, to the best of the author’s knowledge, is the first attempt to classify AD from imaging techniques that measure the alterations of peripheral microcirculation and ANS activity, rather than central nervous modifications, demonstrating in a data-driven approach that AD also produces microcirculatory peripherical modifications.

Concerning the computational efficiency of the proposed method, the computation time was around 3 min, employing a PC with Windows 10pro (Intel(R) Core (TM) i7-7700; CPU @ 3.60 GHz 3.60 GHz; RAM: 16 GB). Hence, the low computation load allows us to employ the model in a not highly performing PC, fostering the employment of the method in outpatient environments; in this aspect, it should be highlighted that specialized operators are not necessary to run the model.

The first limitation of the study concerns the sample size. Further studies should indeed be performed, increasing the number of participants. In fact, since the IRT-based classification proposed relies on multivariate analysis, the outcome of the procedure might highly increase with larger sample numerosity. Of note, the classification outcome was cross-validated with a leave-one-out procedure, which is to train the classifier excluding one subject at a time and test its performance on that subject. Thus, although the sample size could be considered limited, the results obtained are indeed generalizable. It is worth highlighting that, given the small sample numerosity, the leave-one-out cross-validation was preferred in order to train the model employing the largest train sample but further studies are indeed necessary to test the performances of the models employing other methods of cross-validation (e.g., K-fold cross-validation).

Moreover, increasing the sample size may improve the performance of the classifier by decreasing a possible in-sample overfitting effect, which may allow us to consider more ROIs in the analysis and employ more complex machinery, such as deep learning. In addition, advanced SVM classifiers have been proposed to provide more robust and accurate classifications with respect to the canonical SVM [73,74,75]. Hence, further studies should absolutely be performed enlarging the sample size in order to test the capabilities of these SVM advanced algorithms to classify AD patients.

Another limitation of the study is related to the length of the resting state recording (5 min). In fact, this temporal window does not allow for a reliable analysis of the low-frequency bands, such as the metabolic band (0.003–0.02 Hz). Of note, the duration of the recordings was limited to 5 min because of the clinical management of the patients but further studies are indeed necessary to investigate the impairments related to lower frequency ranges; these may be associated with circadian thermoregulatory dysregulations, already observed in AD patients [23,67].

Furthermore, it could be of great interest to foster a multimodal approach for the ANS activity monitoring AD patients during resting state, combining IRT with other techniques such as EEG, electrocardiography, and galvanic skin response. Such a multimodal approach could provide information regarding possible associations between peripheral impairments and central nervous system disruption, in order to provide a more comprehensive investigation of the physiology related to the origin of the symptoms of AD.

In addition, it could be worth investigating the capability of IRT to assess altered microcirculation not only in the facial region but also in other body areas such as the hands. From this perspective, the capability of low-cost IR cameras to discriminate AD from HCs in not-controlled thermal environments should be investigated. In fact, these kinds of cameras could be easily integrated into portable devices (e.g., mobile phones), providing the possibility to perform AD classification directly by the users. Hence, the obtained information could be integrated into an Internet of Things (IoT) framework, supporting telemedicine applications.

The results of this study cannot provide an alternative tool for the early AD diagnosis but they suggest further exploring the IRT diagnostic potentialities and paving the way to possible employment of this technique as a supporting clinical procedure. In fact, IRT is a completely non-invasive, contactless, portable, and relatively cheap technique that could be easily employed in an outpatient environment, with no need of highly specialized operator. These findings show the possibility to evaluate microvascular dysregulation in early AD patients during resting state through IRT. Recordings performed during resting state in a controlled thermal environment allow us to exclude thermoregulatory effects and autonomic responses due to external stimuli (e.g., cognitive tasks) in the analysis. In fact, the novelty of this study relies on the possibility to assess peripheral microcirculation impairments in a contactless manner during the resting state by means of IRT and ML. Finally, since the results obtained are based on a frequency-domain data analysis of the IRT signals, the dysregulation of the microcirculatory system is not highlighted by different superficial temperatures but by modifications of the heat transmission mechanism to the skin due to impaired microcirculatory functionality. Hence, these results foster the investigation of further metrics to analyse microvascular impairments from the temperature time course.

## 5. Conclusions

In this study, vascular impairments in AD patients at rest have been investigated through IRT. The approach based on ML demonstrated a strong sensitivity of the frequency content of the thermal time course of the nose tip to the presence of AD. Specifically, an accuracy of 79.7% and 82.1% were reached for DTC and SVM classifiers respectively. This study opens the possibility of using ML statistical approaches for IRT data analysis, with the aim to improve its diagnostic capabilities to detect AD dementia. The results confirmed that altered microcirculation in early AD patients is detectable through facial IRT during the resting state, suggesting possible employment of this technique in clinical practice for early AD screening. Further studies are indeed necessary to provide effective technological support for AD early detection, which could be easily integrated into an IoT framework for telemedicine applications.

## Figures and Tables

**Table 1:** The *t*-test results concerning the differences between AD and HCs for each PSD at the different frequency bands.

Frequency Band	*t*-Stat	d.f.	*p*-Value
Neurogenic	−0.154	24	0.879
Myogenic	0.029	24	0.977
Respiratory	−1.794	24	0.085
Cardiac	−1.376	24	0.182

**Table 2:** Accuracy, sensitivity, and specificity (expressed as percentage) of the ML classifiers investigated. The best performances are highlighted in bold.

ML Classifier	Accuracy	Sensitivity	Specificity
**KNN**			
Fine	53.8	46.7	63.6
Medium	57.7	80	27.3
Coarse	69.2	86.7	45.5
**Ensemble classifiers**			
Bagged Trees	65.4	66.7	63.6
Subspace Discriminant	61.5	60	63.6
**SVM**			
Linear	61.5	93.3	18.2
Quadratic	69.2	73.3	63.3
Cubic	61.5	73.3	45.5
RBF	79.7	72.7	**86.7**
**DTC**	**82.1**	**90.9**	73.3

## Data Availability

The data presented in this study are available on request from the corresponding author. The data are not publicly available due to privacy issues. The developed models are available at the following link: https://figshare.com/s/37c57a90fe62dd2f21f3 (accessed on 13 September 2022).
